# Crystallographic and DFT study of novel dimethoxybenzene derivatives

**DOI:** 10.1186/s13065-025-01496-0

**Published:** 2025-05-14

**Authors:** Nancy N. Elewa, Ahmed F. Mabied

**Affiliations:** 1 Physics Department, Faculty of Science, Ain Shams University, Cairo, Egypt; 2 X-Ray Crystallography Lab, Solid State Physics Department, National Research Centre, 33 Bohouth Street, Dokki, Cairo, 12622 Egypt

**Keywords:** Dimethoxybenzene, Bioactivity, Crystal structure, Hirshfeld analysis, DFT

## Abstract

Dimethoxybenzene derivatives are versatile compounds with significant pharmaceutical applications. This study investigates the synthesis of two dimethoxybenzene derivatives, focusing on their structural, electronic, and intermolecular interaction properties. Crystallographic analysis showed that the compounds crystallize in the monoclinic system, with planar phenyls, stabilizing their structures by hydrogen bonds and intermolecular interactions. Density Functional Theory (DFT) calculations were employed to analyze electronic properties, including HOMO and LUMO energy levels, energy gaps (E_g_), and molecular electrostatic potentials (MEPs). The study compared (PBE) DFT functional to hybrid functionals PBE0 and B3LYP. The most time-efficient calculation was PBE; however, the one with the lowest total energy was the hybrid functional B3LYP, as the energies were − 172,318.3710 eV and − 33,332.8726 eV for compounds 1 and 2, respectively. The basis set Def2-TZVP produced the lowest energy but required more computation than 6-311G(d,p). The compounds' energy gaps, hardness, and softness values demonstrated their thermodynamic stability, which is particularly advantageous for pharmaceutical applications. The MEPs revealed compound 2 was more electrophilic and a hydrogen bond donor, while compound 1 was more nucleophilic and a strong hydrogen bond acceptor. The study highlights the significance of dimethoxybenzene derivatives as therapeutic materials, paving the way for further research on their various applications.

## Introduction

Dimethoxybenzene derivatives, a class of organic compounds characterized by two methoxy (-OCH_3_) groups attached to a benzene ring, have been found in diverse applications in several fields [1–4]. Different dimethoxybenzene derivatives, such as 1,2-dimethoxybenzene, 1,3-dimethoxybenzene, 3,4-dimethoxybenzene, and 4,5-dimethoxybenzene, are used in many important scientific fields. The specific applications of these compounds hinge upon their isomeric form. Due to their unique chemical and structural features, they are very valuable in pharmaceutical applications because of their powerful antioxidant properties that help eliminate free radicals. Resveratrol, a natural dimethoxybenzene derivative, is found in grapes. Preclinical studies have demonstrated its potential in preventing diseases associated with oxidative stress, including cardiovascular diseases and neurodegenerative disorders [5].

These compounds often serve as key drug intermediates in synthesizing various pharmaceuticals, including anti-inflammatory agents, anti-cancer drugs, and anti-viral medications. In particular, 1,2-dihydroisoquinoline derivatives [4] act as delivery systems that transport drugs through the otherwise highly impermeable blood–brain barrier [5]. These compounds also exhibit sedative [6], antidepressant [7], antitumor, and antimicrobial activities [8]. Isoquinolines manufacture dyes, paints, insecticides, and antifungal agents as a solvent for extracting resins and terpenes and as corrosion inhibitors [9, 10].

They are also used as alternatives to natural products, allowing for the study of structure–activity relationships and the development of improved therapeutic agents. Also, they have critical applications in material science in organic electronics, such as the fabrication of organic light-emitting diodes (OLEDs) and organic solar cells. Their electronic properties and structural characteristics contribute to the performance and efficiency of these devices. These compounds can be incorporated into polymer chains to impart specific properties, such as enhanced conductivity, optical activity, or mechanical strength. They are used to develop materials for electronic devices, coatings, and adhesives. Also, certain dimethoxybenzene derivatives exhibit liquid crystal behavior in liquid crystals, making them valuable components for their unique properties that enable light transmission and polarization control.

In natural product chemistry, dimethoxybenzene derivatives are commonly found in natural products, such as plants and fungi. Researchers isolate and characterize these compounds to understand their biological activities and potential medicinal applications. The biosynthesis pathways of dimethoxybenzene derivatives have been investigated to elucidate the enzymes and intermediates involved in their production by organisms. This knowledge can be applied to synthesizing these compounds in a laboratory setting. Dimethoxybenzene derivatives have shown potential as plant growth regulators or herbicides, aiding in agricultural practices. These compounds can contribute to the flavour and aroma of foods and beverages, and they are used in the fragrance industry to create perfumes and colognes. Dimethoxybenzene derivatives can be used as analytical reagents in various chemical assays and spectroscopic techniques.

Crystallographic analysis of single crystals provides essential insights into molecular geometry, stereochemistry, and molecular interactions with targets [6–12]. Hirshfeld surface (HS) analysis is a powerful tool in crystallography for visualizing and quantifying intermolecular interactions within a crystal lattice. It provides a detailed understanding of the spatial distribution of electron density and the nature of interactions between molecules, which are critical for elucidating the stability, packing, and reactivity of crystalline materials [13]. Density functional theory (DFT) calculations are used to determine binding affinities and interactions with the target, including geometry optimization, molecular electrostatic potential (MEP), and frontier molecular orbital (FOs) analysis of compounds' electronic properties and energies [14–16]. The paper emphasizes the importance of choosing functionals and basis sets for accurate and efficient calculations [17].

In conclusion, dimethoxybenzene derivatives have demonstrated their versatility and importance in various applications. Their unique properties, including antibiotic and antioxidant activity, electronic characteristics, and natural product occurrence, have made them valuable compounds in different fields, such as pharmaceuticals, materials science, and natural product chemistry. Continued research in this area is expected to uncover new and exciting applications for these versatile molecules.

Even though many studies have documented the pharmacological activities of dimethoxybenzene derivatives, there is a limitation in systematic evaluations and comparisons across various derivatives and their mechanisms of action in drug discovery. The primary research gap in the pharmaceutical applications of dimethoxybenzene derivatives is the need for a comprehensive analysis of the novel derivatives [18]. This study presents the synthesis, crystallographic characterization, and computational analysis of two novel dimethoxybenzene derivatives. Single-crystal X-ray diffraction was employed to determine their molecular structures, while Hirshfeld surface analysis was used to elucidate intermolecular interactions. Density functional theory calculations were conducted to explore the electronic properties, including molecular electrostatic potential (MEP) and frontier molecular orbital (FMO) analyses, to elucidate these compounds' unique hydrogen bonding patterns, thermodynamic stability, and reactivity. Specifically, we reveal how substituting bromine and methoxy groups influences the electronic properties and intermolecular interactions, offering a deeper understanding of their potential in pharmaceutical applications. By comparing different DFT functionals and basis sets, we also provide a methodological advancement in predicting the properties of such derivatives. These findings pave the way for the targeted design of dimethoxybenzene-based compounds with optimized bioactivity and stability, addressing gaps in existing studies.

## Experimental

### Synthesis

**Scheme 1:**
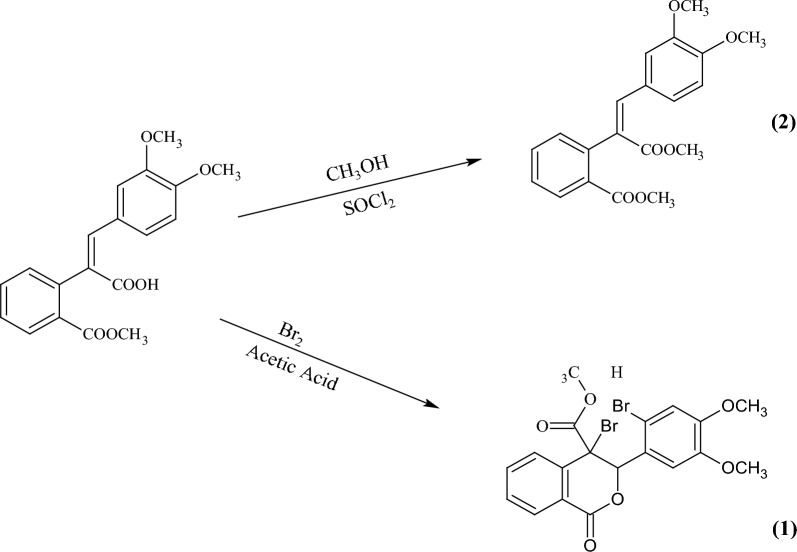
Synthesis of the title compounds

The first compound synthesized from the solution of (2*Z*)-3-(3,4-methoxyphenyl)-2-[2-(methoxycarbonyl) phenyl] acrylic acid, which was prepared by putting 2 gm of it in 30 mL of absolute methanol at room temperature, then SOCl_2_ (5 mL) was added dropwise to the solution while stirring at such a rate as to maintain the room temperature for 30 min. Then, the reaction was refluxed in a water bath for eight hours. A colourless microcrystal of methyl 2-[(Z)-2-(3, 4- dimethoxyphenyl)-1 (methoxycarbonyl) vinyl] benzoate, (**1**), was formed after cooling, having molecular formula C_20_H_20_O_6_, with a yield of 86%. Then, it is recrystallized from acetone at 40–45 ˚C by slow evaporation technique to obtain colourless cube-shaped single crystals after a week (scheme 1).

Also, the second compound, methyl 4-bromo-3-(2- bromo-4,5-methoxyphenyl)-1-oxo-3,4-dihydro-1H-isochromene-4-carboxylate (2), was prepared from the solution of methyl 4-bromo-3-(2-bromo-4,5-dimethoxyphenyl) -1-oxo-3,4- dihydro—1H—isochrone – 4-carboxylate that was prepared by putting 1 gm of it in 10 mL of acetic acid at room temperature. Also, the bromine solution was prepared by adding 1 mL of bromine to 10 mL of acetic acid. Then it was added dropwise to a (2*Z*)-3-(3,4-dimethoxyphenyl)-2-[2-(methoxycarbonyl) phenyl] acrylic acid solution while stirring at room temperature for 30 min. The reaction mixture was left at room temperature (25–30 ˚C) overnight and then diluted with cold water. A buff precipitate was observed, filtered off, and washed with water to get the compound with molecular formula C_19_H_16_Br_2_O_6_ yielding 89% (scheme 1).

The compounds were prepared according to the reported procedures [19, 20]. For recrystallization, the obtained material is dissolved in a mixture of petroleum ether (60:80), ethanol, and acetone. Slow evaporation took one week to get colourless, plateless, single, good crystals.

### X-ray data collection

X-ray single crystal diffraction data were collected from a Kappa CCD Enraf Nonius diffractometer with MoK_α_ radiation [21]. The data reduction was performed using Denzo and Scalepak [22]. The maXus [23] program suite and CRYSTALS package [24] were employed to resolve and refine the structures. The analysis and graphical display of the structure were conducted using the crystallographic software PLATON [25], Mercury [26], and ORTEP-3 [27] for Windows. The complete crystallographic data set is provided in the supplementary material, along with the structures deposited at the Cambridge Crystallographic Data Centre with CCDC 2365007 and CCDC 2365008 for structures 1 and 2, respectively.

### NMR spectroscopy

The ^1^H and ^13^C NMR spectra were obtained using a VARIAN Gemini at 300 MHz and a Jeol at 75 MHz, respectively, in Palo Alto, California. The chemical shifts of the molecule are referenced using tetramethylsilane (TMS) as an internal standard.

### Molecular computations

The molecular geometry of the studied compounds in their ground state was optimized using density functional theory, which employed different functionals and basis sets. All calculations were performed using the ORCA package and the Avogadro visualization program [28, 29].

## Results and discussions

### NMR spectroscopy

Table 1 shows the estimated structure and the corresponding NMR results. For compound 2, its ^1^H NMR spectrum showed the presence of multiple signals at δ 6.7 – 8.1 ppm characteristic of seven aromatic protons and six proton signals in the aliphatic region at δ 3.83 characterizing the methyl group (OCH_3_), and two singlet signals at 3.89 and 3.68 ppm which are characteristic of COOCH_3_. For compound 1, all the aliphatic region signals for CH_3_ are found except the one at 3.89 ppm, as that group was cycled upon bromine addition, resulting in a new distinctive signal at 6.67 ppm. Also, for compound 1, the proton NMR spectra showed only six aromatic protons for the multiple signals at δ 6.6 – 8.1 ppm, confirming the replacement with the Br ion.

**Table 1:** The estimated structure and the corresponding ^1^H and ^13^C NMR spectra for both compounds

Compound 1	Compound 2
	
	
	

^13^C NMR spectrum of both compounds was similar except for a single extra methyl signal at δ 52.5 ppm for compound 2 and two additional single peaks at 64.4 ppm and 86.6 ppm that were found in compound 1 spectra characterizing the aliphatic carbon C and CH, respectively

It can be concluded that compounds 1 and 2 have different NMR spectra, especially in the aromatic and aliphatic regions, which suggests that their hydrogen bonding patterns are different. As the chemical shifts in compound 1 range from 7.40 to 7.79 ppm, it seems likely that aromatic protons are involved in hydrogen bonding. Compound 2, on the other hand, has chemical shifts that range from 8.74 to 9.99 ppm, which means that its deshielding effects are stronger. This could be because it has more hydrogen bonds or different electronic environments. These changes in hydrogen bonding could affect the stability of molecules and how they interact with biological targets, which is an important thing to consider in pharmaceutical applications. For example, compound 2's stronger hydrogen bonds may make it more likely to bind to certain receptors, making it a better drug candidate. Further analysis of these patterns could provide insights into optimizing the compounds for therapeutic use.

### Crystal structure analysis

**Fig. 1:**
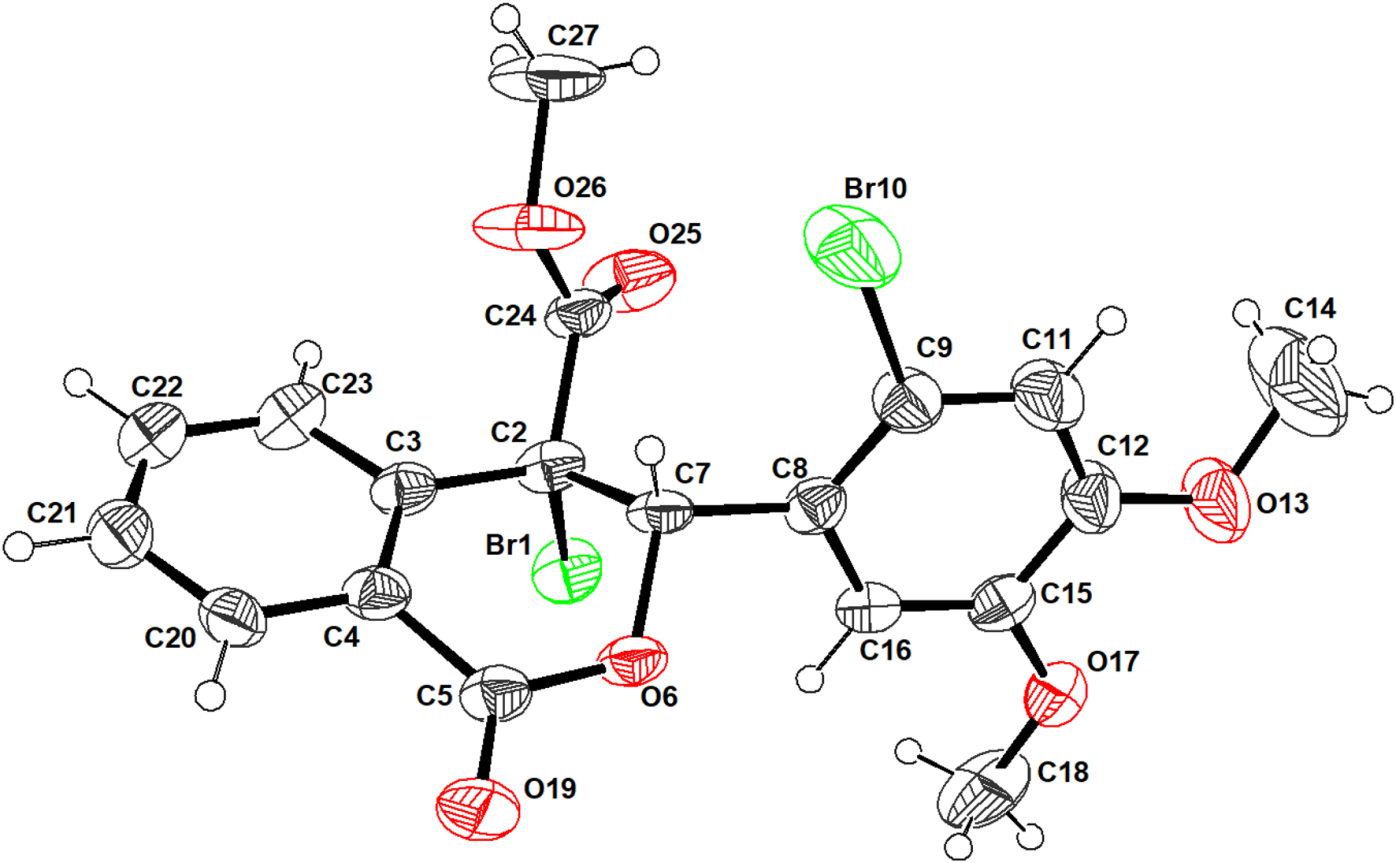
An ORTEP view of compound 1, with displacement ellipsoids drawn at the 50% probability level, showing atom numbering

**Fig. 2:**
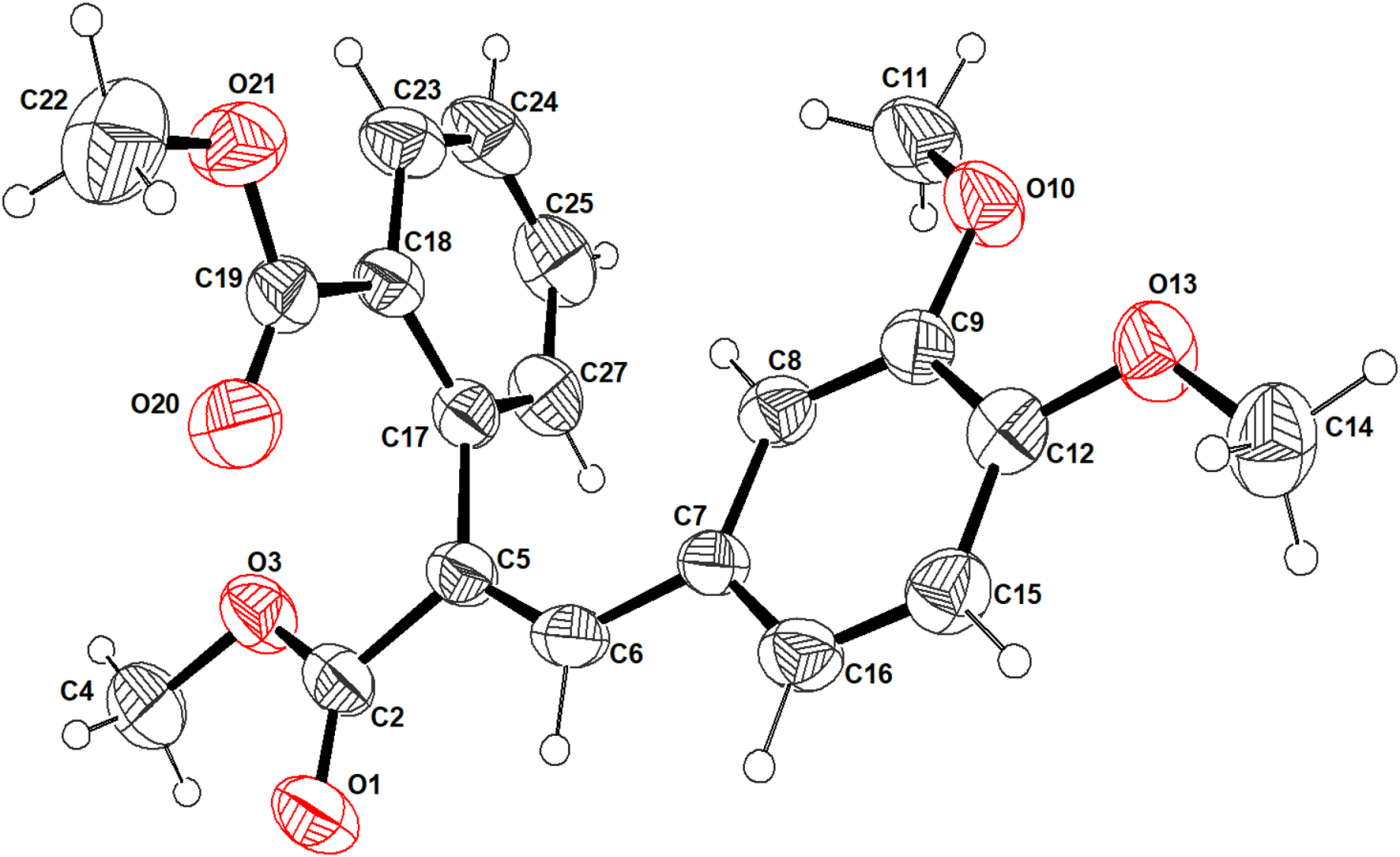
An ORTEP view of compound 2, with displacement ellipsoids drawn at the 50% probability level, showing atom numbering

Figures 1 and 2 present the crystal structures of compounds 1 and 2, respectively, from an ORTEP perspective. Compounds 1 and 2 crystallize in the monoclinic system, with four molecules in the unit cell for compound 1 and two molecules for compound 8. The P2_1_/a and P2_1_ space groups were identified for compounds 1 and 2, respectively.

**Fig. 3:**
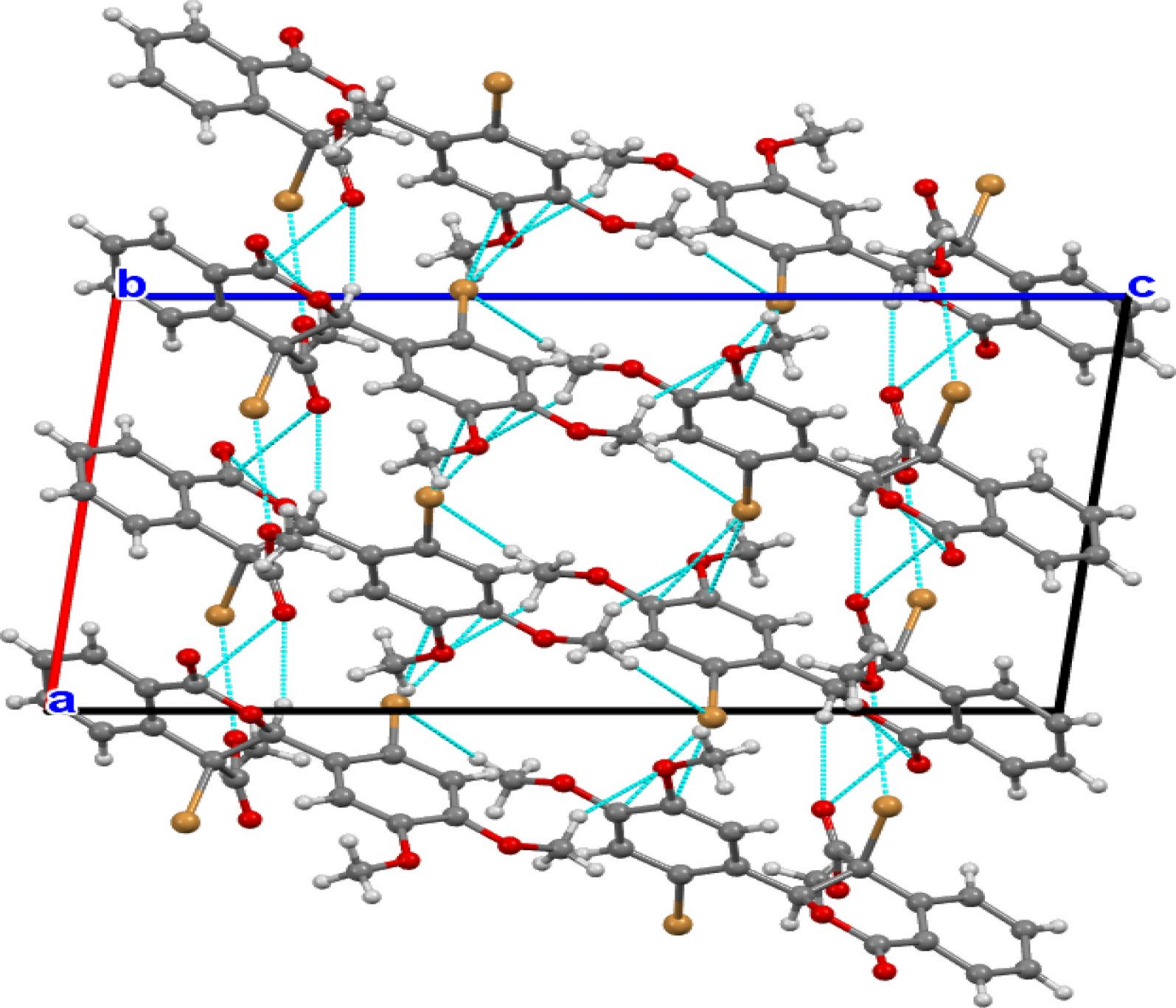
A view of the packing diagram for compound 1 along the **b-axis**. Hydrogen bond contacts with dashed blue lines

The two phenyl moieties in both compounds are planar. A least-squares plane calculation reveals that the said atoms lie in a plane. The torsion angles further support the planarity of the biphenyl rings. The packing diagram of compound 1 is shown in Fig. 3. A network of intermolecular contacts that stabilize the structure with different symmetry codes is shown in Fig. 3. The intermolecular hydrogen bonds and angles are shown in Table 2.

**Table 2:** Selected hydrogen-bond parameters of compound 1

*D*—H···*A*	*D*—H (Å)	H···*A* (Å)	*D*···*A* (Å)	*D*—H···*A* (°)
C7—H71···O25^i^	0.95	2.45	3.222 (11)	138
C7—H71···Br10	0.95	2.6200	3.207(6)	120.00
C16—H161···Br1	0.95	2.8700	3.329(6)	111.00

Symmetry code(s): (i) *x* + 1/2, -*y* + 3/2, *z*

**Fig. 4:**
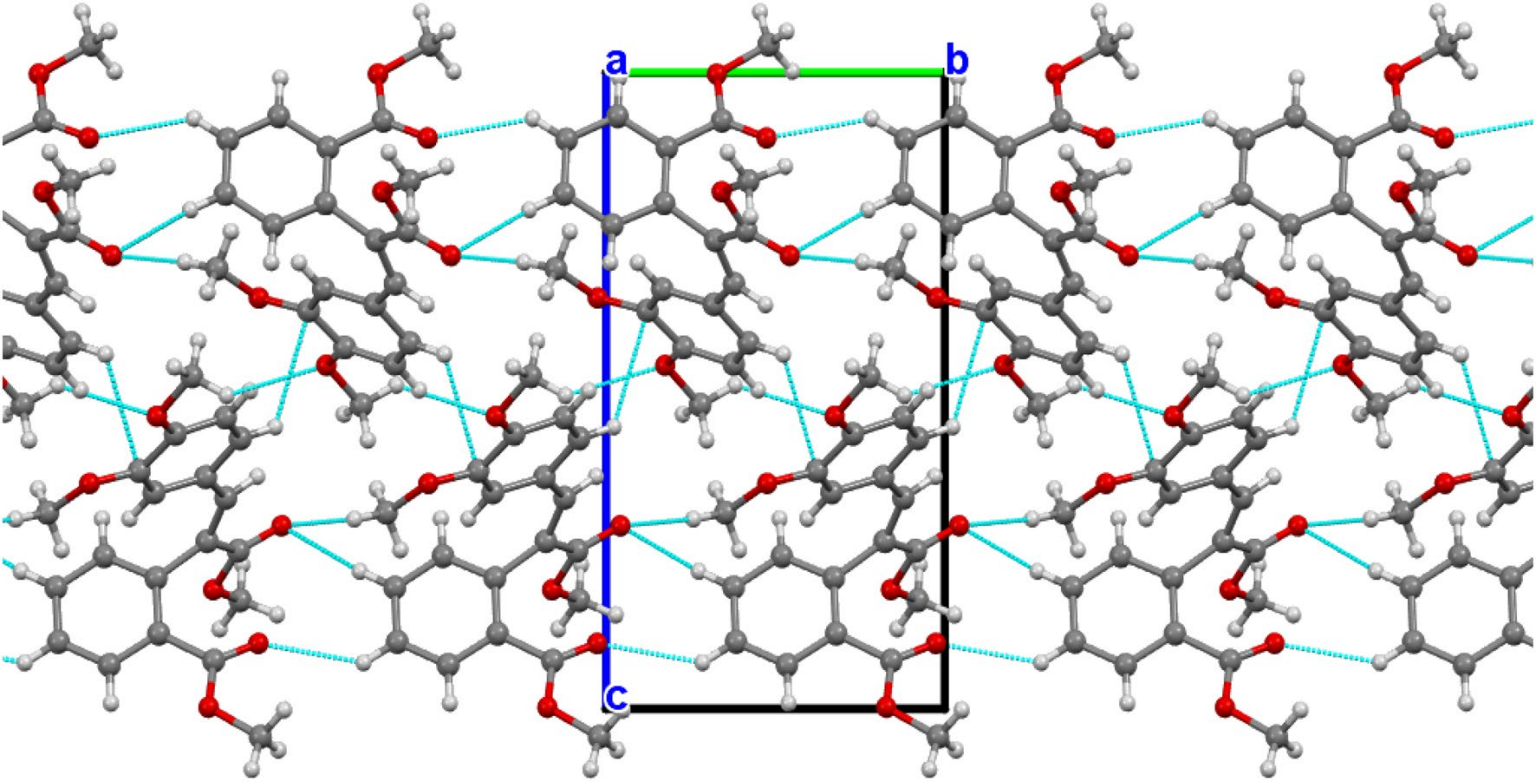
A view of the packing diagram for compound 2 along **a-axis**. Hydrogen bond contacts with dashed blue lines

The packing diagram of compound 2 is shown in Fig. 4. A network of intermolecular contacts stabilizes the structure with different symmetry codes, as shown in Fig. 4. The intermolecular hydrogen bonds and angles are shown in Table 2. The dimethoxybenzene group is present in the two compounds, although it is presented in compound 1 as Bromo dimethoxybenzene. According to the numbering scheme shown in Figs. 1 and 2, the bond lengths of the Bromo dimethoxybenzene and dimethoxybenzene moieties for compounds 1 and 2, respectively, can be found in the supplementary materials.

Intermolecular interactions, particularly hydrogen bonds, played a significant role in stabilizing the crystal packing. For compound 1, key hydrogen bonds included C7—H71···O25 and C16—H161···Br1, while compound 2 exhibited interactions such as C8—H81···C17 and C25—H251···O1. Hirshfeld surface analysis further complemented the crystallographic data, providing insights into the intermolecular contacts and their contributions to the crystal stability. The analysis highlighted the importance of van der Waals interactions and hydrogen bonding in the packing arrangements. These structural insights, combined with density functional theory calculations, elucidated the electronic properties and reactivity of the compounds, offering a deeper understanding of their potential applications in pharmaceuticals and materials science (Table 3).

**Table 3:** Selected hydrogen-bond parameters of compound 2

*D*—H···*A*	*D*—H (Å)	H···*A* (Å)	*D*···*A* (Å)	*D*—H···*A* (°)
C8—H81···C17	0.95	2.51	3.143 (10)	124
C14—H142···O13^i^	0.95	2.58	3.345 (10)	137
C24—H241···O20^ii^	0.95	2.56	3.329 (10)	139
C25—H251···O1^ii^	0.95	2.54	3.464 (10)	163

Symmetry code(s): (i) -*x*-1, *y* + 1/2, -*z* + 1; (ii) *x*, *y*-1, *z*

### Hirshfeld analysis

**Fig. 5:**
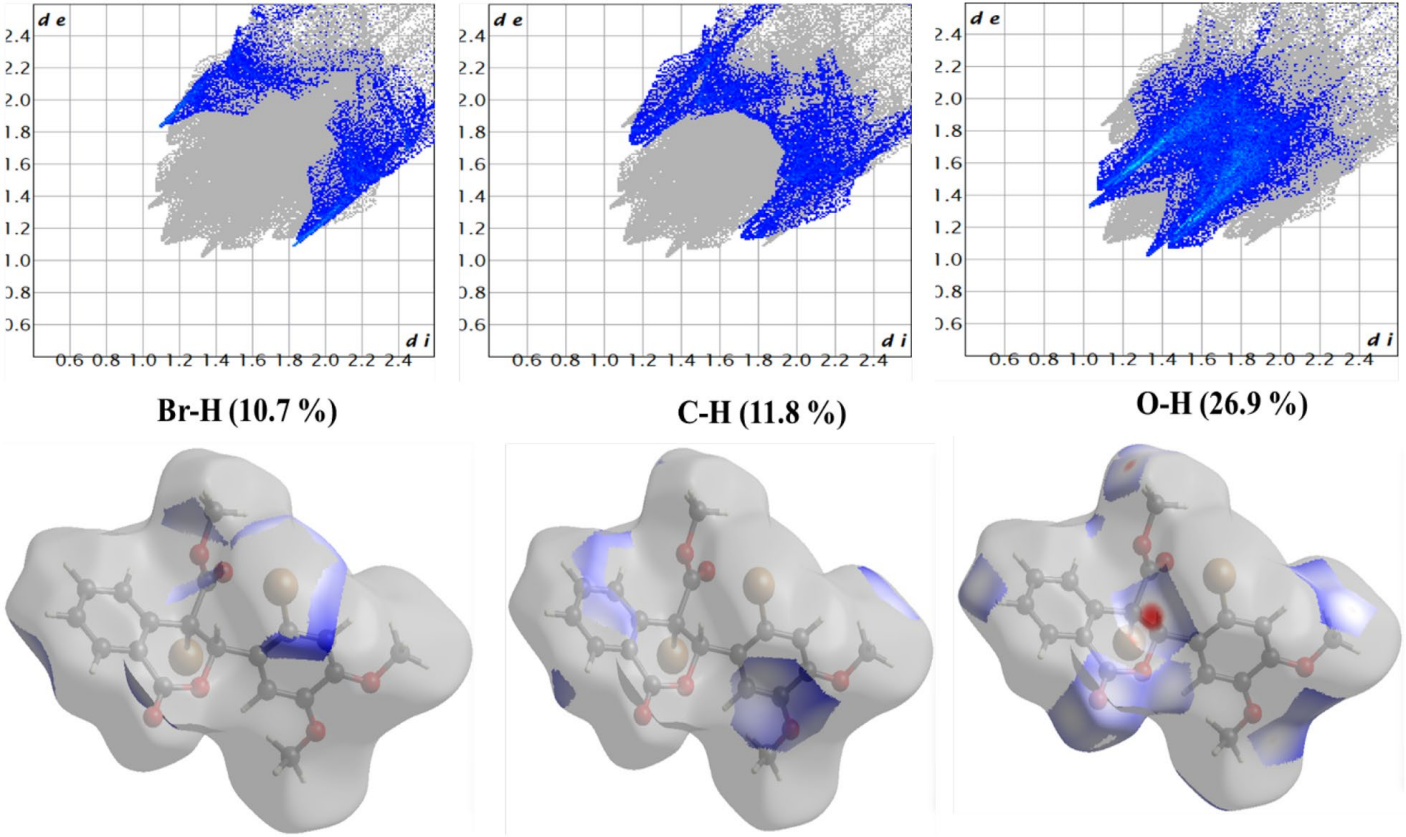
Hirshfeld surface results of compound 1 showing the d_norm_ plot and 2D fingerprint for the relative contributions. The red colour represents shorter contacts, blue for longer contacts, and white for contacts close to *d*_norm_ equal zero

**Fig. 6:**
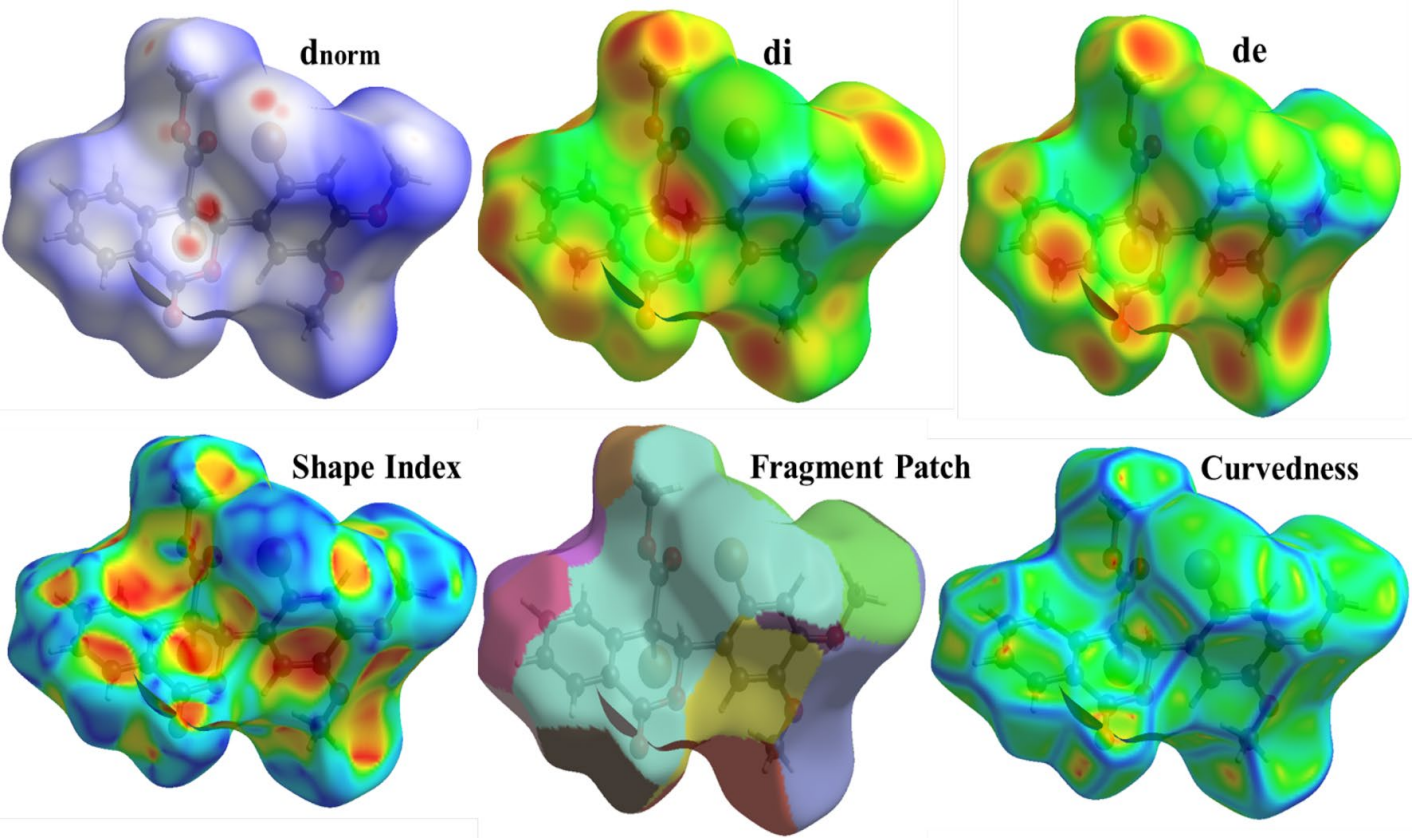
Hirshfeld surfaces of compound 1

**Fig. 7:**
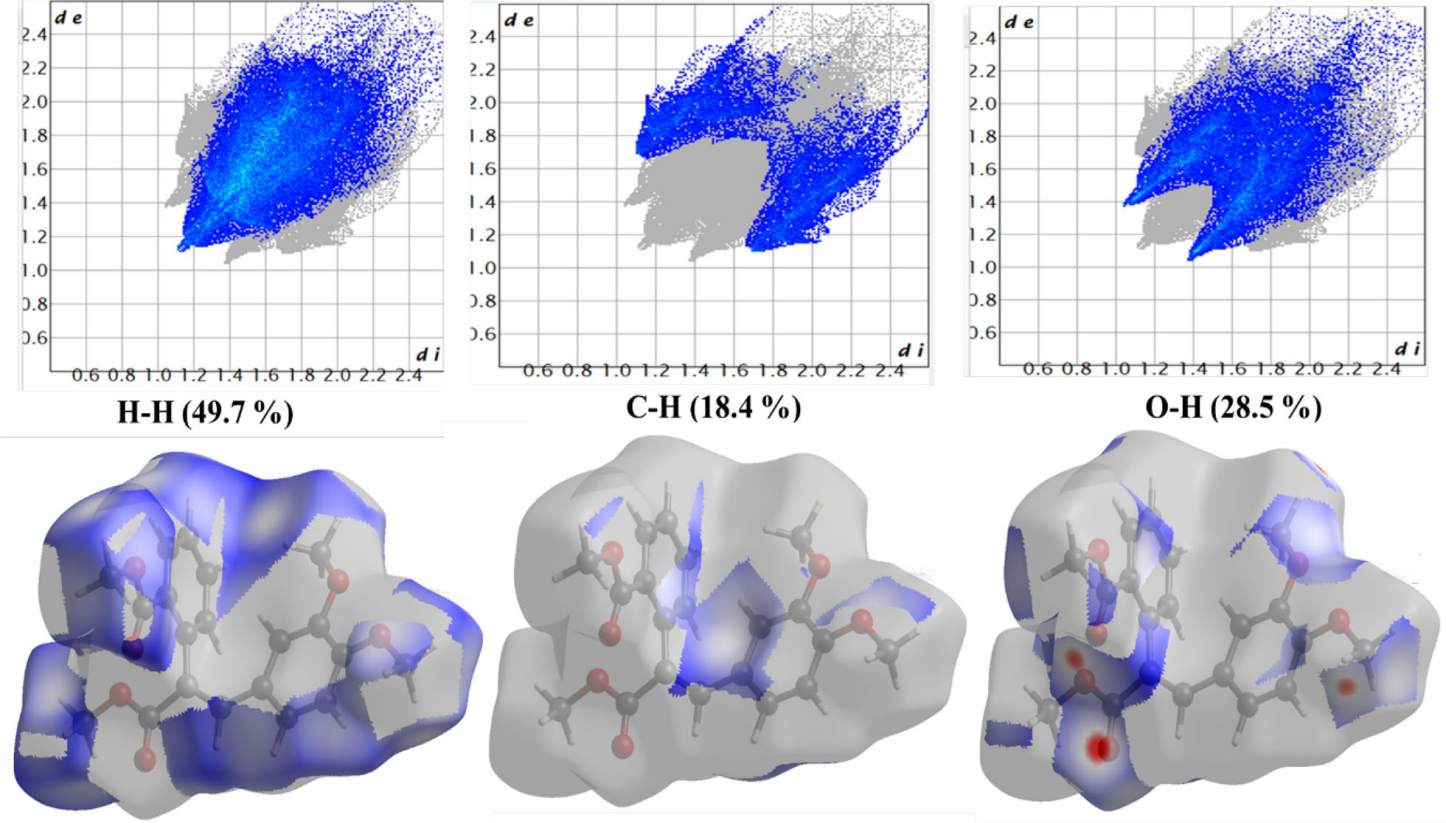
Hirshfeld surface results of compound 2 showing the d_norm_ plot and 2D fingerprint for the relative contributions. The red colour represents shorter contacts, blue for longer contacts, and white for contacts close to *d*_norm_ equal zero

**Fig. 8:**
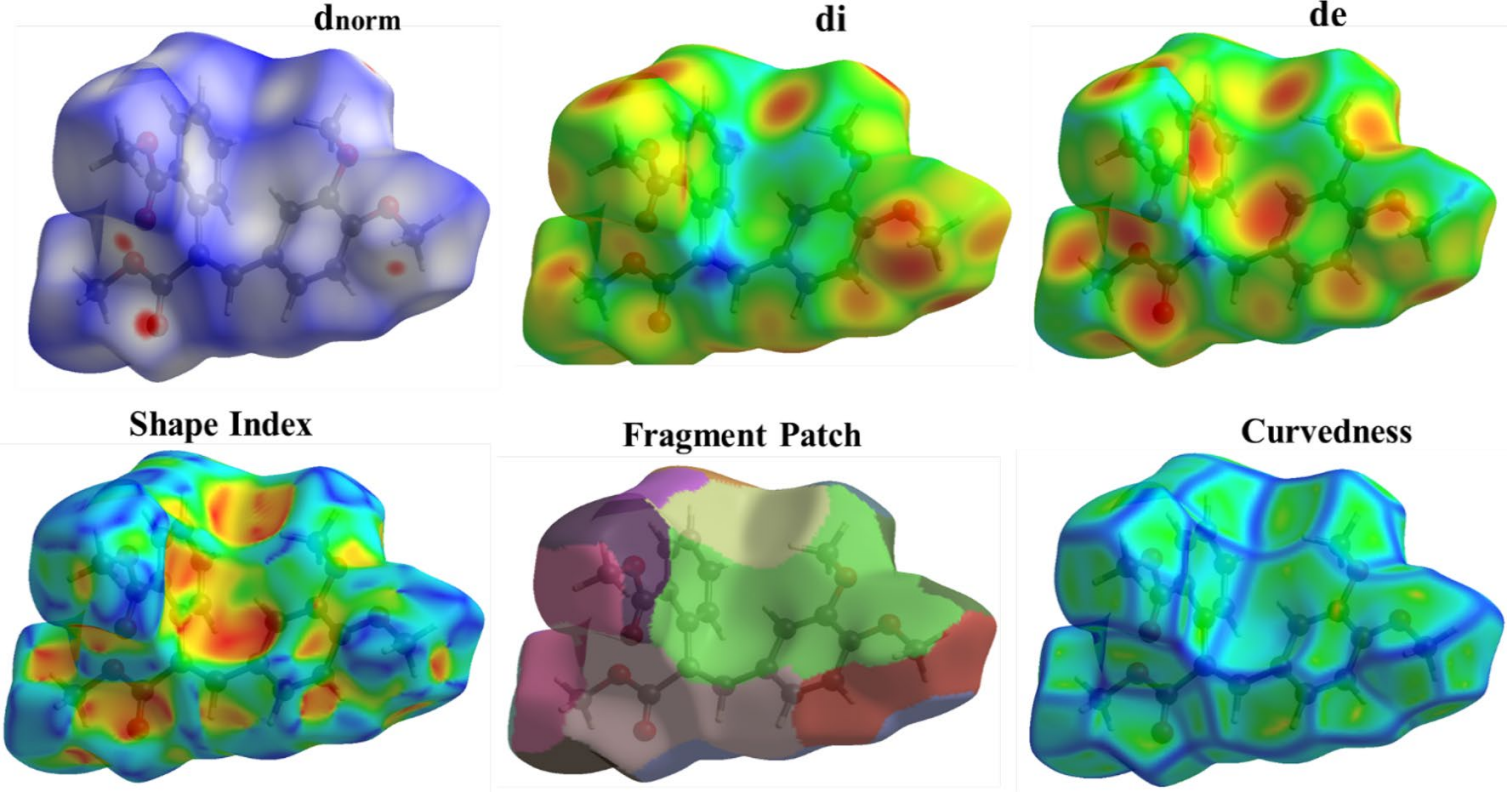
Hirshfeld surfaces of compound 2

The weight function for each atom in the molecule is represented by the Hirshfeld surface (HS), which is described in terms of charge density. The entire number of atoms in the molecule (promolecule) divided by the total number of atoms in the crystal (procrystal) yields this. The Hirshfeld surface analysis conducted in this study provides a detailed visualization and quantification of intermolecular interactions within the crystal structures of the synthesized dimethoxybenzene derivatives [13, 30]. By mapping the normalized contact distance (d_norm_), the analysis highlights regions of close intermolecular contacts, with red areas indicating shorter contacts and blue areas representing more extended contacts. The 2D fingerprint plots derived from the Hirshfeld surfaces offer a comprehensive breakdown of the relative contributions of different interactions, such as hydrogen bonding, van der Waals forces, and halogen interactions, to the overall crystal packing. Figures 5 and 6 show the results obtained; compound 1 reveals significant hydrogen bonding interactions, particularly involving the bromine and oxygen atoms, stabilizing the crystal structure. Similarly, for compound 2, the Hirshfeld surface analysis underscores the importance of C-H···O and C-H···C interactions in maintaining structural integrity, as shown in Figs. 7 and 8. These findings complement the crystallographic data and provide a deeper understanding of molecular packing and stability. These are crucial for predicting these compounds' physicochemical properties and potential applications in pharmaceuticals and materials science. The integration of Hirshfeld surface analysis with DFT calculations further enhances the interpretation of the electronic and structural characteristics of the dimethoxybenzene derivatives, offering valuable insights for future research and development.

Intermolecular interactions have a great impact on stability and reactivity [31]. The crystal structures of compounds 1 and 2 play a crucial role in determining their stability and reactivity, which are essential for their potential pharmaceutical applications. The hydrogen bonds C—H…O and C—H···Br in compound 1 and compound 2 like bonds in addition to C—H···C, reinforcing structural rigidity. Moreover, halogen bonding and π-π stacking between aromatic rings contribute to the thermodynamic stability of the crystals [31]. Hirshfeld surface analysis shows compound 1 has more hydrogen bond interactions due to its brominated structure, while compound 2 has a broader distribution of hydrogen bond contacts due to its steric bulk. This may influence its reactivity by shielding electrophilic sites. carbonyl groups at compound 1 are more accessible for nucleophilic attack, aligning with its higher hydrogen bond acceptor capacity. Implications for reactivity and drug design include the hydrogen bond donor/acceptor balance in compound 2 (nucleophilic carbonyls) vs. compound 1 (electrophilic bromine sites). This suggests that compound 2 is more likely to engage in polar interactions with biological targets. In contrast, compound 1's halogen bonds could enhance binding to hydrophobic pockets. Methoxy groups in both compounds facilitate intramolecular charge delocalization, reducing electrostatic repulsion and enhancing lattice energy. However, compound 1's bromine substituents introduce steric constraints that slightly destabilize the crystal, offset by stronger halogen bonding.

### Molecular computations

Density Functional Theory employs various functionals and basis sets to analyze the properties of dimethoxybenzene derivatives [32]. The choice of functional significantly impacts the accuracy of electronic property predictions, such as HOMO and LUMO energy levels. The literature reveals that B3LYP and PBE are two of the most commonly used functionals [33–35]. Each has exhibited different performance characteristics in predicting molecular geometries and reactivity. Drug designers commonly use the two functionals mentioned above for ligands. The hybrid functional B3LYP is preferred over the PBE because it is more accurate and costs less to compute [32, 36, 37].

**Fig. 9:**
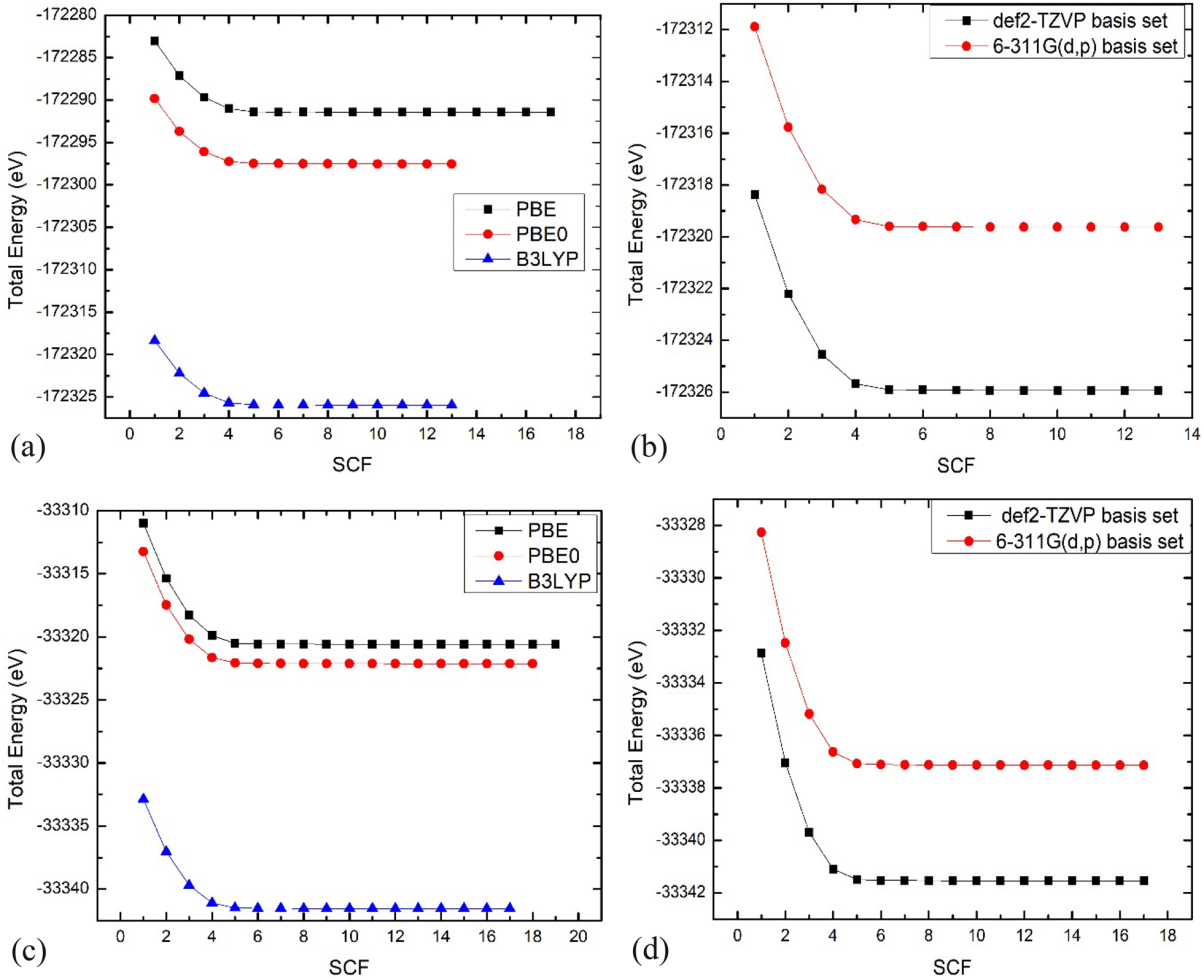
The total energy convergence graph for the 2 compounds: compound 1 (a)and {b) and compound 2 (c) and (d) with different functionals and basis sets

This study compared the pure DFT functional Perdew-Burke-Ernzerhof (PBE) GGA to hybrid functionals PBE0 and B3LYP [38, 39]. The PBE and B3LYP [19, 20] compare the optimum structure with the lowest energy and convergence rate for the different functionals. We have chosen tight convergence criteria for the geometry optimizations, as the energy change's convergence criteria is 10^–8^ Hartree. The B3LYP functional has generally been used for compounds with H-bonding interactions. However, the most time-efficient calculation was the one done with PBE; however, the one that gave the lowest total energy was the hybrid functional B3LYP, as the energies were − 172,318.3710 eV and − 33,332.8726 eV for compound 1 and compound 2, respectively (Table 4). In addition, we studied the effect of the basis sets on the calculation's accuracy and time, as shown in Fig. 9. Although the basis set Def2-TZVP gave the lowest energies in the two compounds, it was more expensive than the 6-31G(d,p) basis set, as it took more time for convergence.

**Table 4:** The lowest energy for optimized structure for both compounds with different functional and basis sets

COMPOUND 1	Functional/basis sets	Energy (E_h_)	Energy (eV)	SCF convergence
	PBE_ def2-TZVP	− 6331.28610	− 172,283.0535	17
	PBE0_ def2-TZVP	− 6331.53464	− 172,289.8166	13
	B3LYP_ def2-TZVP	− 6332.58400	− 172,318.3710	13
	B3LYP_ 6-311G(d,p)	− 6332.34567	− 172,311.8859	13
COMPOUND 2	Functional/basis sets	Energy (E_h_)	Energy (eV)	SCF convergence
	PBE_ def2-TZVP	− 1224.31749	− 33,315.3727	19
	PBE0_ def2-TZVP	− 1224.23899	− 33,313.2366	18
	B3LYP_ def2-TZVP	− 1224.96060	− 33,332.8726	17
	B3LYP_ 6-311G(d,p)	− 1225.11720	− 33,337.1337	17

**Fig. 10:**
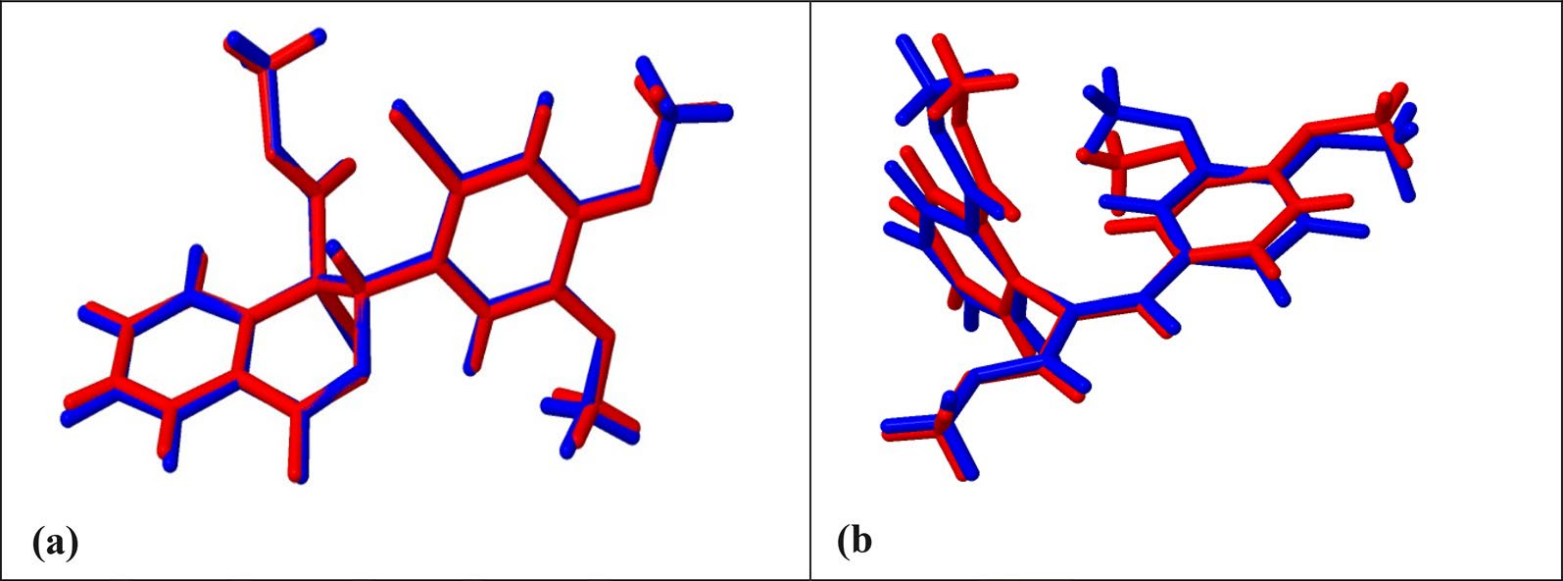
Overlay view of the X-ray (red) and DFT structure (blue) for compound 1 (a) and compound 2 (b) computed by B3LYP/Def2-TZVP method

It can be concluded that the B3LYP functional, known for its reliability in hydrogen-bonded systems, delivered the lowest total energy for both compounds, proving its correctness for this study. However, the PBE function was praised for its processing efficiency. The basis set Def2-TZVP produced the lowest energy but required more computation than 6-311G (d,p) (Fig. 10).

In summary, the B3LYP functional was preferred over PBE because it better predicted the electronic properties and non-covalent interactions important for the dimethoxybenzene derivatives examined. Even though B3LYP takes more time to run, it gives us a better balance between accuracy and cost when compared to PBE. The Def2-TZVP basis set was picked over 6-311G(d,p) because it describes dispersion interactions better and is more accurate overall.

The HOMO (highest occupied molecular orbital) and LUMO (lowest unoccupied molecular orbital) energies are very popular quantum chemical descriptors that describe the reactivity; hence, calculating the HOMO and LUMO gap is essential in estimating the chemical reactivity and stability of the molecule and the electrons transfer between the electron-accepting LUMO and the electron-donating HOMO [40, 41].

For each of the compounds, hardness (η), softness (S), and energy gap were calculated from the energies of HOMO and LUMO [42, 43]. Hardness (η) is defined as the resistance of a molecule to charge transfer, calculated as half the energy gap between the LUMO and HOMO. Softness (S) is the inverse of hardness, representing the ease of charge transfer. They are calculated using the following equations: Eg = [LUMO − HUMO]; η = [LUMO − HUMO]/2; S = 1/η.

**Fig.11:**
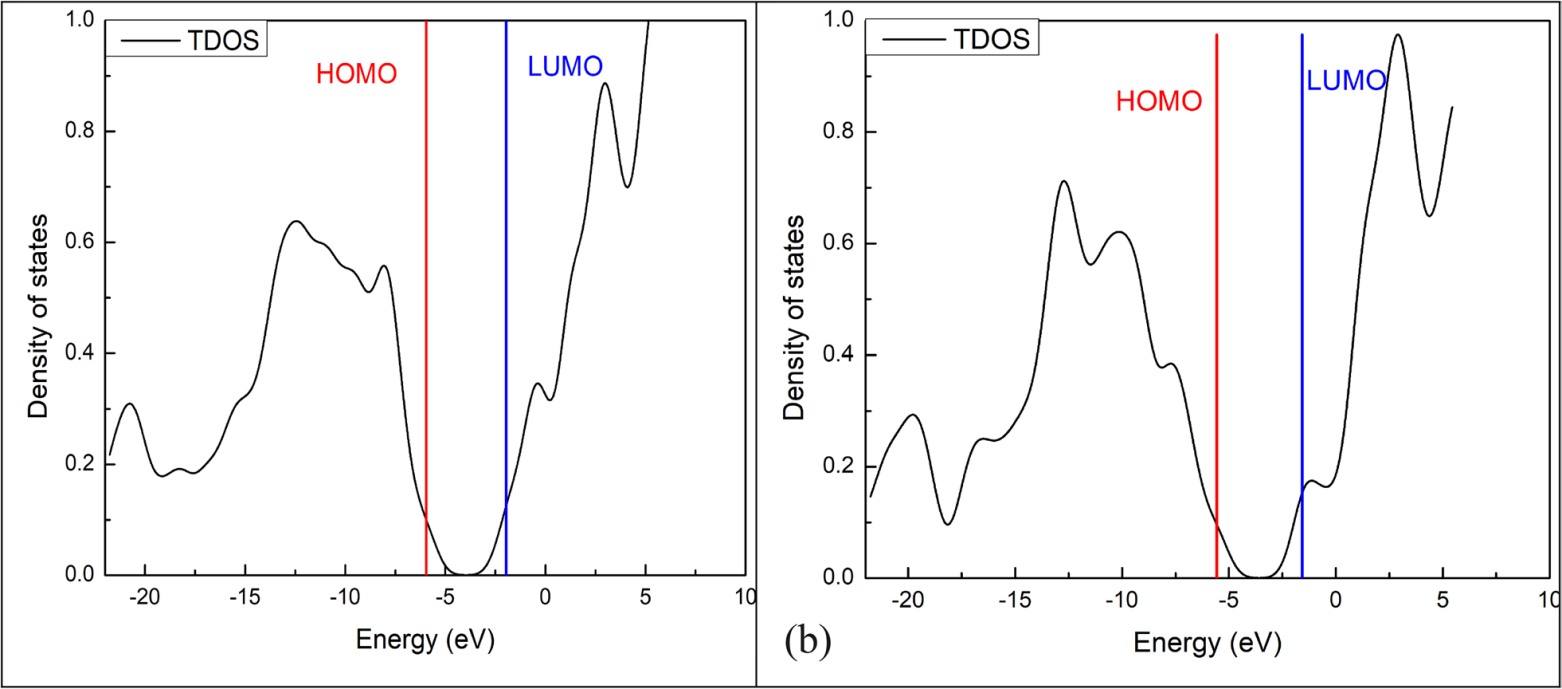
The total density of states for compound 1(a) and compound 2(b) with the energy gap indicated as computed by the B3LYP/def2-TZVP method.

As shown in Table 5, Figs.  11and12, the compounds have very close values. These values suggest that both compounds exhibit excellent thermodynamic stability, influenced by their electronegativity and molecular electrostatic potential maps. The energy gap values indicate excellent thermodynamic stability for the molecules, introducing them as stable pharmaceutical agents [44]. Both compounds' energy gaps indicate chemical stability, while compound 2's slightly lower energy gap (3.98 eV) suggests greater reactivity, consistent with its electrophilic MEP regions.

**Table 5:** Energy (eV) of HOMO and LUMO, energy gap, hardness, and softness of compound 1 and compound 2

Name	HOMO (eV)	LUMO (eV)	Eg (eV)s	hardness (η)	softness (S)
Compound 1	− 5.9636	− 1.9621	4.0015	2.0008	0.4998
Compound 2	− 5.5665	− 1.5769	3.9895	1.9948	0.5013

**Fig. 12:**
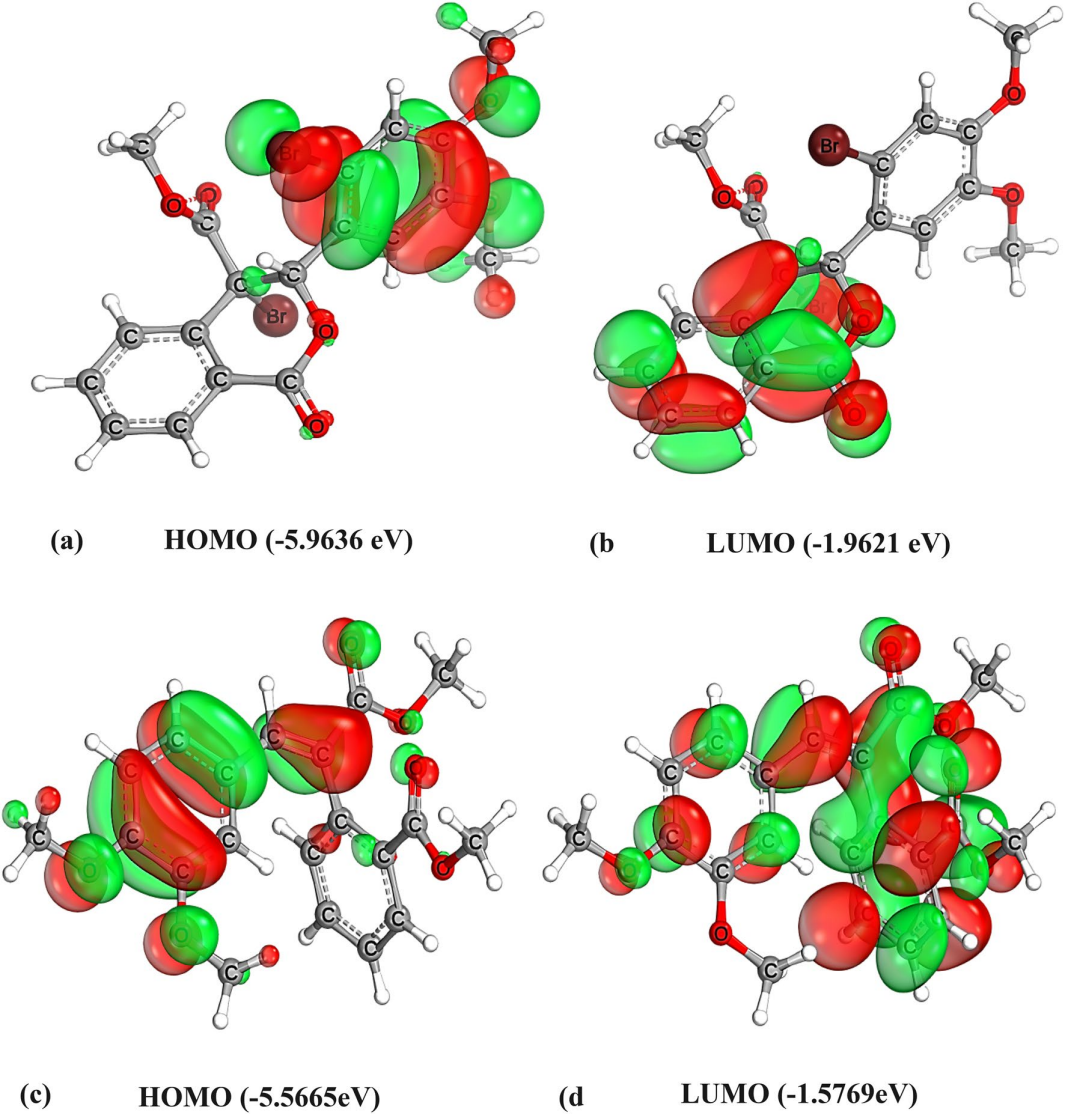
Molecular orbital surfaces and energy levels are given in parentheses for the HOMO and LUMO of compounds 1 (a,b) and 2 (c,d) computed by the B3LYP/def2-TZVP method

The electron affinity (A) and the ionization potential can be estimated from the HOMO and LUMO energy values. The electron affinity (A) = Negative of the LUMO energy, and the ionization potential (I) = Negative of the HOMO energy [43].

The isotropic theoretical shielding for compounds 1 and 2 was compared to the experimental chemical shifts (in ppm) using B3LYP for the two basis sets, def2-TZVP and 6-311G(d,p). The average isotropic magnetic shielding tensor for each ^1^H and ^13^C nucleus σ_cal_ was calculated. Then, the isotropic chemical shifts δ_cal_ was defined as δ_cal_ = σ_TMS_ − σ_cal_, where σ_TMS_ is the isotropic shielding constant of ^1^H and ^13^C in the reference sample used, trimethylsilane (TMS).

Some statistical parameters, such as root mean square error **RMSE** and regression correlation factor** R**^**2**^ are calculated and tabulated in Table 6. Among all statistical descriptors, both parameters are vital in validating the theoretical chemical shifts to those experimentally measured [45, 46]. The DFT method for estimating the ^1^H and ^13^C NMR chemical shifts is considered more accurate the smaller the RMSE parameter is.

**Table 6:** Statistical parameters indicate the agreement of the calculated chemical shielding and the experimental chemical shift for both compounds using B3YLP with two basis sets: def2-TZVP and 6-311G(d,p)

		Regression correlation coefficient **R**^**2**^		Root mean square error **(RMSE)**	
		^13^C	^1^H	^13^C	^1^H
**Compound 1**	def2-TZVP	0.961	0.992	13.289	3.600
	6-311G(d,p)	0.962	0.989	12.962	3.804
**Compound 2**	def2-TZVP	0.715	0.811	33.02931	2.64256
	6-311G(d,p)	0.733	0.805	32.35096	2.655753

**Fig. 13:**
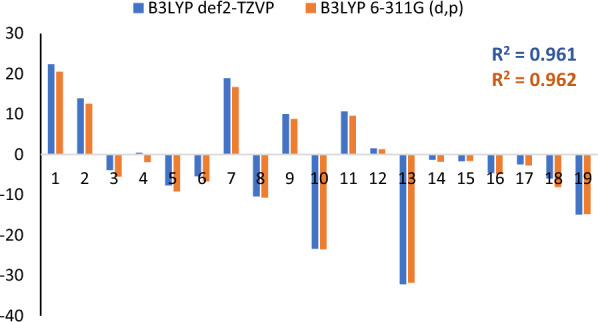
The difference between the experimental chemical and theoretical isotropic shielding (δ) for ^13^C NMR for compound 1 with the blue and red regions indicates the def2-TZVP and 6-311G(d,p) basis sets, respectively

The difference (error) between experimental and calculated chemical shifts is shown in Fig. 13 and is calculated as:

$$ \delta \, = \,\delta_{cal} {-}\delta_{exp} $$

$$\text{RMSE }=\sqrt{\frac{1}{N}\sum {\updelta }^{2}}$$

A good linear regression correlation coefficient (R^2^ > 0.96) is obtained for compound 1 (Fig. 13), however the agreement is less obvious for compound 2. The less correlated shielding between observed and theoretical calculated for compound 2 could be attributed to the anisotropy effect.

### Molecular electrostatic potential (MEP)

**Fig. 14:**
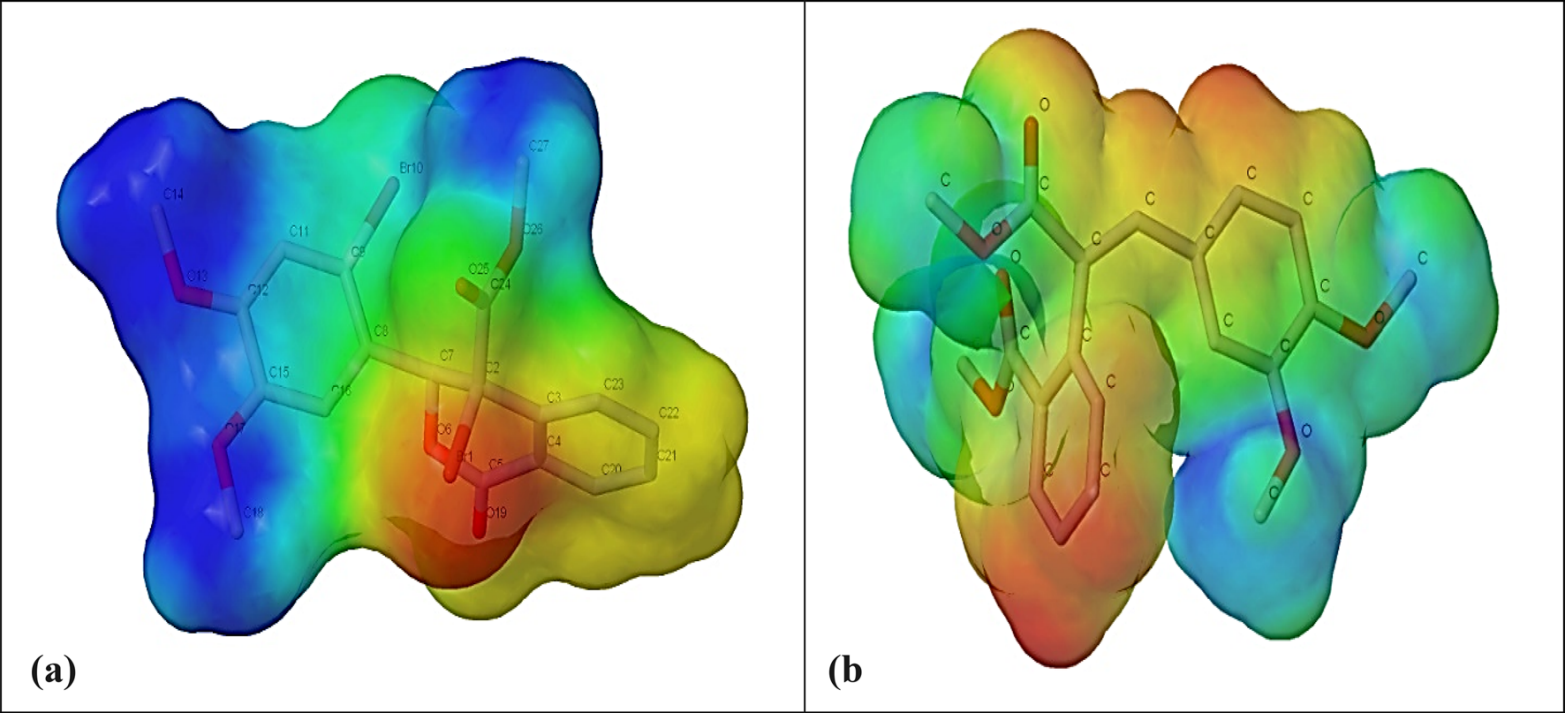
The molecular electrostatic potential for compound 1(a) and compound 2(b) with the red regions indicates the more electronegativity sites

MEP is the pictorial method for identifying the reactive sites of active pharmaceutical ingredients towards positively or negatively charged reactants, allowing them to predict the hydrogen bonding and, hence, the structure–activity relationships of the molecule [47, 48]. It is a good tool for understanding the distribution of electron density in space around the molecule by mapping the total density surface on the electrostatic potential energy surface, depicting the size, shape, charge density, and reactive sites of the molecules [49]. The expression gives the molecular electrostatic potential V(r) produced due to the combined effect of positive and negative charges across the molecule corresponding to electrons and nuclei.

$$ V\left( r \right) = {\text{~}}\mathop \sum \limits_{A} \frac{{Z_{A} }}{{\left( {\overrightarrow {{R_{A} }}  - {\text{~}}\vec{r}} \right)}} - {\text{~}}\smallint \frac{{\rho \left( {\overrightarrow {{r'}} } \right)}}{{\left( {\overrightarrow {{r'}}  - {\text{~}}\vec{r}{\text{~}}} \right)}} $$

where Z_A_ is the charge of the nucleus A, found at distance R_A_, and ρ(r′) is the electronic density function of the electrostatic potential indicated by a colour code, where red indicates the highest negative potential, blue indicates the highest positive potential, and green indicates the zero-potential region. Red < yellow < green < blue is the increasing order of the potential distribution on the MEP map. In compound 1, the red region near the carbonyl groups (C = O) indicates areas of the highest negative potential, signifying regions rich in electron density. These are the most likely sites for electrophilic attack or hydrogen bond acceptance. The blue areas around the methoxy groups (-OCH₃) show places with the highest positive potential, meaning no more electrons exist. These are the most likely sites for nucleophilic attack or hydrogen bond donation. The benzene ring, bromine, and the parts of the molecule, which are defined by green colours, indicate a more neutral electrostatic potential. The MEP suggests that the carbonyl groups are the most likely sites for the electrophilic attack, while the methoxy groups are prone to nucleophilic attack, as a hydrogen bonding donor. However, the electrostatic potential map for compound 2 shows significant electronegative (red) regions, primarily around the oxygen atoms in the hydroxyl groups. This suggests these locations are highly susceptible to electrophilic attack and are strong hydrogen bond acceptors. The less electronegative regions (green/yellow) indicate less reactive areas, as shown in Fig. 14.

In summary, compound 2 is likely more reactive in multiple locations toward electrophiles and can engage in more dispersed hydrogen bonding. However, compound 1 reacts more specifically, favouring electrophilic attack at a particular site and being more likely to act as a hydrogen bond donor in the blue regions. These differences will significantly influence how these compounds interact with other molecules and biological targets and their chemical behaviour in different environments.

The structure–activity relationships (SAR) of dimethoxybenzene compounds are influenced by the position and number of methoxy groups and other substituents, which modulate their interactions with biological targets and their physicochemical properties [50–52].

SAR of the studied dimethoxybenzene derivatives is influenced by their molecular geometry, intermolecular interactions, and electronic properties, which crystallographic and Hirshfeld surface analyses could reveal. Hydrogen bonds and other intermolecular interactions stabilize the crystal structures of both compounds. These interactions can be correlated with their potential interactions with biological targets. Hirshfeld surface analysis further highlights that compound 1 exhibits significant hydrogen bonding involving bromine and oxygen atoms, while compound 2 shows strong C-H···O interactions. These interactions suggest that the position and nature of substituents (e.g., methoxy and bromo groups) modulate the compounds' ability to form hydrogen bonds, which is critical for binding to biological targets. The electronic properties, including HOMO–LUMO energy gaps and molecular electrostatic potentials (MEPs), also provide insights into reactivity and stability. Compound 2, with more electrophilic regions around oxygen atoms, is likely to engage in stronger hydrogen bonding as an acceptor, while compound 1, with nucleophilic sites, may act as a donor. These differences in electronic distribution and interaction potential can influence the compounds' binding affinity and specificity toward biological targets, guiding their optimization for pharmaceutical applications.

## Conclusion

This study investigated the structural and electronic properties of two dimethoxybenzene derivatives through crystallographic analysis and density functional theory (DFT) calculations. The compounds crystallized in the monoclinic system, with planar phenyl moieties stabilized by intermolecular hydrogen bonds and van der Waals interactions. Hirshfeld surface analysis revealed significant intermolecular contacts, with compound 1 exhibiting strong hydrogen bonding involving bromine and oxygen atoms, while compound 2 showed prominent C-H–-O interactions. DFT calculations revealed that the hybrid functional B3LYP provided the lowest total energy, while the PBE functional was more time-efficient. DFT calculations, particularly using the B3LYP functional, provided insights into the electronic properties, including HOMO–LUMO energy gaps and molecular electrostatic potentials (MEPs), indicating excellent thermodynamic stability and reactivity. The MEP maps identified electrophilic and nucleophilic sites, suggesting potential drug design and material science applications. These findings underscore the importance of intermolecular interactions and electronic properties in determining the stability and reactivity of dimethoxybenzene derivatives, paving the way for their further exploration in pharmaceutical and material applications.

## Data Availability

The datasets used and/or analyzed during the current study are available from the corresponding authors upon reasonable request.
